# High-performance liquid chromatography and Enzyme-Linked Immunosorbent Assay techniques for detection and quantification of aflatoxin B_1_ in feed samples: a comparative study

**DOI:** 10.1186/s13104-019-4538-z

**Published:** 2019-08-07

**Authors:** Achenef Melaku Beyene, Xiangwei Du, Dwayne E. Schrunk, Steve Ensley, Wilson K. Rumbeiha

**Affiliations:** 1 College of Veterinary Medicine and Animal Sciences, University of Gondar, Gondar, Ethiopia; 2 College of Veterinary Medicine, Iowa State University, Ames, IA USA

**Keywords:** Aflatoxin B_1_, ELISA, Feed, HPLC

## Abstract

**Objective:**

Comparison was done between high-performance liquid chromatography (HPLC) and a competitive enzyme-linked immunosorbent assay (ELISA) for detection and quantification of aflatoxin B_1_ (AFB_1_) in feed samples. The two procedures were standardized and validated before the actual experiment. Five concentrations (0, 5, 10, 20 and 30 ppb) of feed samples were used for both methods. For the HPLC technique, the samples were extracted in acetonitrile/water (90/10) solution, cleaned-up using solid phase extraction (SPE) column, and derivatized by water/trifluoroacetic acid/glacial acetic acid (35/10/5) solution before instrument analysis. The samples were extracted in 70% methanol for the ELISA technique.

**Results:**

The two tests showed very strong linearity with correlation coefficient value of > 0.99 using standard solutions. The mean recovery rate was 92.42% (with relative standard deviation (RSD) of 5.97) and 75.64% (RSD = 34.88) for HPLC and ELISA, respectively. There was no statistically significant difference in recovery rate between the two methods. There was a positive correlation (r = 0.84) between them which indicated that the two techniques can be used to detect and quantify aflatoxin B_1_ in feed samples. However, there were variations among replicates for the ELISA method, which shows that this method is more applicable for screening purposes.

## Introduction

Aflatoxins are produced by *Aspergillus* species of fungi as secondary metabolites and found in four main forms (aflatoxin B_1_, B_2_, G_1_ and G_2_) in contaminated grains. Of these, aflatoxin B_1_ is the most common and potent toxin. It accounts for about 75% of all aflatoxin contamination of food and feed in the world. Aflatoxins are polycyclic, unsaturated and highly reactive compounds with relatively high molecular weight [1, 2].

Aflatoxins have multiple negative effects on the health of both human and animals. The toxicity of aflatoxins depends mainly on the amount consumed which may range from acute sickness or death to chronic problems [3, 4]. They can induce stunted growth in young [5] and fertility reduction in adults [6, 7]. Aflatoxins can also undermine child nutrition and development (cognitive and physical) since they interfere with absorption and metabolism of vitamins A and D, iron, selenium, and zinc, and induces protein malnutrition, or kwashiorkor [6, 8]. They are carcinogenic, mutagenic, teratogenic and immunosuppressive in most mammalian species. In addition to the direct health consequences, these toxins have multiple economic impacts which are expressed in terms of loss of human and animal life, increased human and animal health costs, reduced livestock production, disposal of contaminated food and feed, and investment in research and applications to reduce severity of the mycotoxin problems [4]. These toxins also limit the export of food or feed items since different countries have regulations restricting importation of contaminated food or feed [5, 9, 10].

The aflatoxin producing fungi can grow and contaminate a variety of food items including maize, peanuts, corn, sorghum, rice, cassava, cotton seed, millet, wheat and different spices. The aflatoxins can be produced by the fungal growth before harvesting, at harvest, or during storage and processing of grains. Environmental factors such as temperature, drought and relative humidity could potentially favor the development of the fungus and subsequent production of the toxins [9, 11–13].

Due to strict rules and regulations, food and feed producing companies are highly interested in more rapid, accurate, sensitive, simple and cheap techniques for aflatoxin analyses [1, 14] as they need to monitor their products routinely to ensure that aflatoxins are below regulatory limits. Different techniques are available to detect and quantify these toxins in feed and animal products. Although HPLC and ELISA techniques have been utilized for a number of years [14], the available information on comparison of the techniques is limited. In this study, a competitive ELISA method was standardized, validated and its performance was compared with HPLC technique using new method of clean-up steps for detection and quantification of the aflatoxin B1 in feed samples.

## Main text

### Materials and methods

The feed sample (corn) was obtained from Trilogy analytical laboratory (Washington, MO, USA). The reference standard of aflatoxin B1 was purchased from Sigma-Aldrich (St-Louis, MO, USA). The stock standard solution was prepared by dissolving the pre-weighed standard in methanol. A 10 mg portion of the neat standard was dissolved in methanol for a stock solution concentration of 1 mg/mL. The stock solution was proportionally diluted to get the necessary working concentrations of 10 μg/mL and 1 μg/mL. All laboratory procedures were conducted in Toxicology and Nutrition Laboratory at Iowa State University, USA and all solutions and reagents used were of analytical (HPLC) and certified grades. Each procedure was repeated at least three times and results were averaged to enhance precision and accuracy.

### High performance liquid chromatography

#### Sample preparation

The samples were processed based on the technique described by Sinha [14] with some modifications adopted by the laboratory with the objective of minimizing cost without compromising the quality of the results following strict validation procedures. This is the gold standard method used routinely by the laboratory for analysis of aflatoxins in feed. Generally, the sample was extracted, cleaned-up, derivatized and injected into HPLC for detection and quantification.

#### Extraction

Five gram of control feed samples were weighed and added into five 50 mL plastic conical centrifuge tubes. The first tube was without aflatoxin standard (0 ppb), the other tubes were spiked to get 5, 10, 20 and 30 ppb concentrations of Aflatoxin B1 and 20 mL of acetonitrile–water (90:10) solution [14] was added in each tube, vortexed for 10 min and centrifuged at 1895*g* for 5 min. The top layer was filtered through Whatman no 1 filter paper into clean and separate labelled 50 mL plastic centrifuge tubes.

#### Clean-up

For clean-up, 6 mL of extracted sample was passed through a solid phase extraction (SPE) column by gravity which had 1.5 g of 50:50 (w/w) Alumina Neutral/Octadecyl (C18) that was sandwiched between two filter discs. The filtrate of each sample was collected in a labeled 7 mL vial and 4 mL of the filtrate was taken from each vial separately and dried under gentle nitrogen stream at room temperature.

#### Derivatization

The residue obtained in the previous step were reconstituted in 400 μL (35/10/5) (v/v/v) water/trifluoroacetic acid/glacial acetic acid solution [15] vortexed for 10 s at maximum speed and then heated at 65 °C in heating block for 15 min. The solutions obtained from this step were incubated for at least for 20 h at room temperature before HPLC analysis.

#### HPLC and its conditions

HPLC (Waters 2695 separation module) fitted with a vacuum degasser, a quaternary pump, an automatic sample injector, a Waters 2475 Multi-wavelength fluorescence detector and software to control the instrument, data acquisition, and data analysis was used for separation and quantification of aflatoxin B1. The prepared sample was injected automatically and an injection volume of 20 μL was used. The mobile phase consisting of water and acetonitrile was pumped at a flow rate of 1.0 mL/min. A total run time in the HPLC was 33 min.

### Enzyme Linked Immunosorbent Assay

A competitive Enzyme Linked Immunosorbent Assay (ELISA) was performed using the Aflatoxin ELISA test kit r-biopharm AG, Darmstadt, Germany. The procedure was based on the manufacturer’s instructions. Five grams of feed samples were weighed into five 50 mL tubes. As in HPLC procedure, the first tube was blank (0 ppb), the other tubes were spiked to get 5, 10, 20 and 30 ppb concentrations of Aflatoxin B_1_ and 25 mL of 70% methanol was added in each tube and shaken vigorously for 5 min using multitube shaker. The extract was filtered through Whatman No. 1 filter paper and 1.0 mL of the filtrate was diluted with 1.0 mL of deionized distilled water and 50 μL of the diluted filtrate was transferred to each well for analysis. In separate wells, 50 μL of standards were added and 50 μL of enzyme was added to each well. After that, 50 μL of anti-aflatoxin antibody solution was added to each well. It was mixed gently by shaking the plate manually and incubated for 10 min at room temperature. After the incubation, the liquid was dumped out of the wells and by holding upside-down micro-well was tapped onto a clean filter towel to remove all remaining liquid from the wells. The wells were filled with 250 μL of washing buffer and wells were emptied again. Then, 100 μL of substrate was added to each well and mixed gently by shaking the plate manually and incubated for 5 min at room temperature in the dark. Then, stop solution (100 μL) was added to each well and the plate was mixed gently. Finally, the absorbance was measured at 450 nm using microplate reader.

### Result interpretation and data analysis

For the HPLC method, the calibration curve was established by plotting the fluorescence intensity versus the injected concentration of standard by linear regression. The concentration of the aflatoxins in feed sample was calculated based on the standard curve [16, 17]. For the ELISA assay, the amount was calculated automatically by the calibrated microplate reader. The recovery rate was calculated by dividing the amount obtained from HPLC or ELISA by the concentration spiked in a feed sample and multiplied by 100 to express it in percentage. *T* test was used to compare the results of the two test methods.

### Results and discussion

**Fig. 1:**
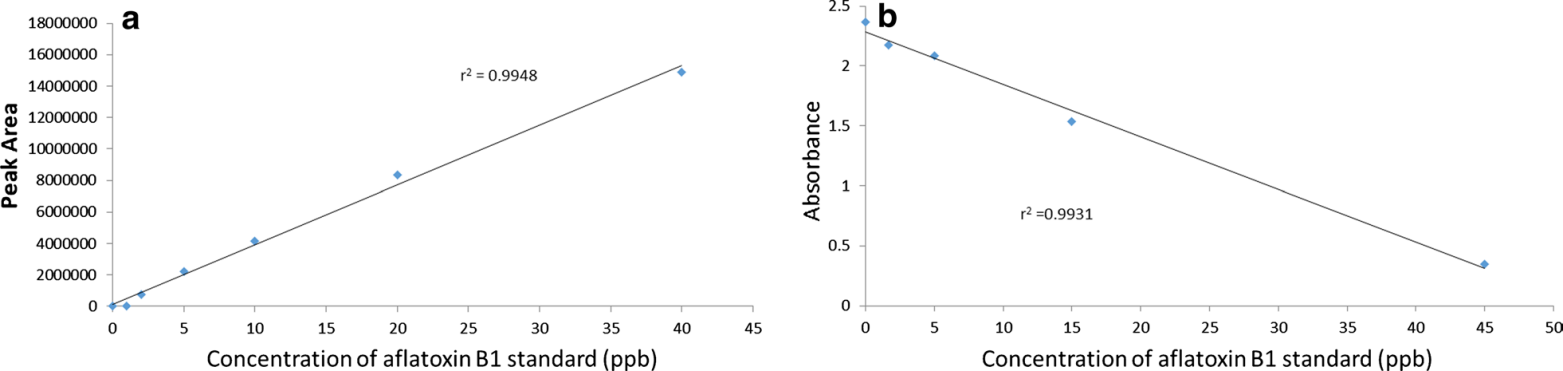
Calibration curve for HPLC (**a**) and ELISA (**b**)

All analytical methods require calibration for quantitation to get reliable and accurate results. Calibration is a process that relates the measured analytical signal to the concentration of analyte [18]. The two techniques used in this study were calibrated before actual experimental samples and showed very strong linearity with a correlation coefficient value of > 0.99 (Fig. 1).

**Fig. 2:**
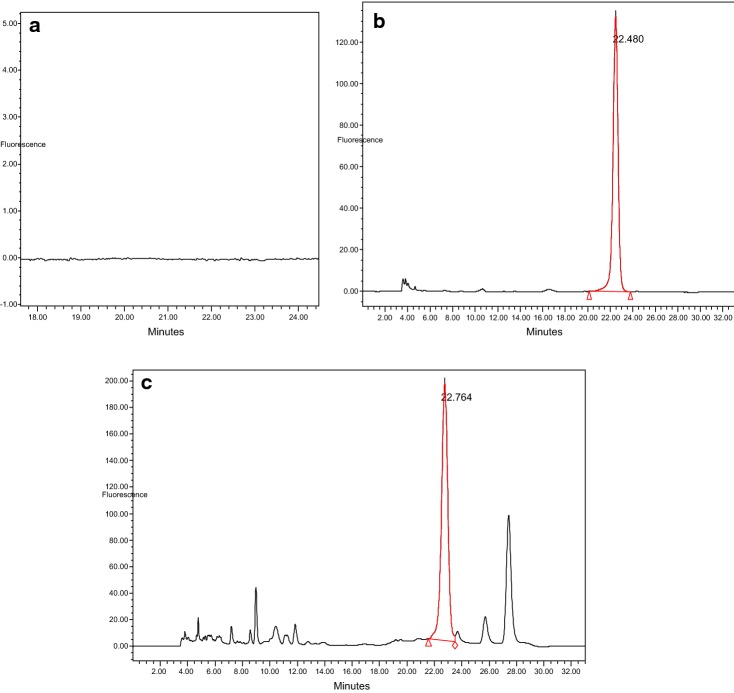
HPLC chromatograms without aflatoxin (**a**), standard (**b**) and spiked feed sample at 10 ppb (**c**)

Figure 2 shows the HPLC chromatograms without aflatoxin, standard and spiked feed sample at 10 ppb. Distinct peak of the Aflatoxin B1 was observed at a retention time 22.5 min.

Chromatographic techniques commonly utilize a clean-up step by using either solid phase extraction (SPE) or immunoaffinity column or both [19]. In this study, a SPE cartridge with Alumina Neutral/C18 sandwiched between two filter discs was used as the clean-up procedure. Although immunoaffinity columns helped to avoid interfering compounds and allow quantification of aflatoxins at very low concentrations [20]. This study demonstrated the feasibility of using SPE. This procedure is relatively cheaper than the immunoaffinity columns; this helps to minimize the cost incurred during HPLC procedure.

The ELISA method is based on the ability of a specific antibody to distinguish the three-dimensional structure of a specific aflatoxin. The competitive ELISA method is commonly used for aflatoxin analysis [19]. A conventional microtiter plate ELISA requires equilibrium of the antibody–antigen reaction. This technique is more rapid and simpler to use than HPLC method. The results can be obtained within 2 to 4 h whereas HPLC takes 2 to 3 days and utilizes several chemicals and purification steps. The chromatographic techniques also require trained skilled technicians and expensive apparatus or equipment [21]. However, the HPLC has high precision, selectivity, and sensitivity [14].

**Fig. 3:**
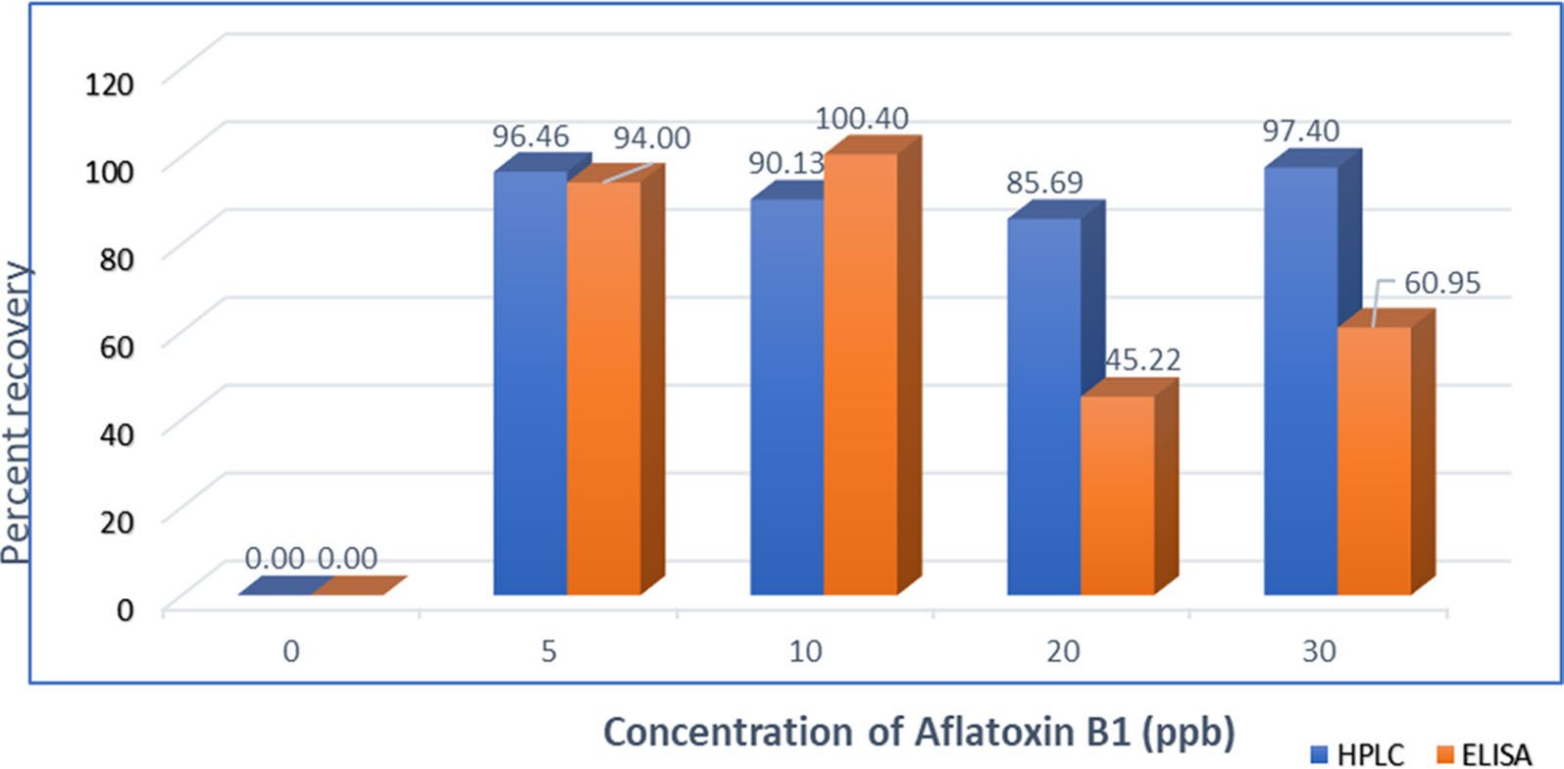
Average recovery of aflatoxin B1 by HPLC and ELISA method

The recovery percentage ranged from 86 to 96%, with mean and relative standard deviation (RSD) values of 92.42 and 5.97%, respectively for HPLC (Fig. 3). The mean recoveries of 85 and 87% were reported by Daradimos et al. [16]. Another report showed the recovery rate ranged from 89 to 109% [22]. For the ELISA technique, recovery was between 45 and 100% with average and RSD of 75.64 and 34.88%, respectively (Fig. 3). There were variations among replicates in ELISA method. This was also reported by Nilufer and Boyacoglu [23]. The percent recovery rate of HPLC was higher and more stable than the percent recovery rate obtained using ELISA. However, there was no significant difference in recovery rate between the two methods and there was a positive correlation (r = 0.84) between two methods. Positive correlation between ELISA and HPLC in feed was also reported by Rossi et al. [24]. Indicating its potential for aflatoxin screening in feed samples. Similarly, Dimitrieska-Stojkovi et al. [25] used ELISA as a screening method whereas HPLC as the confirmatory method.

It has been confirmed that the two tests were able to detect the minimum concentration of aflatoxins set by the Food and Agricultural Organization of the United Nations/World Health Organization (FAO/WHO) standard (15 ppb) and the European Union limit (4–15 ppb) [19, 26–28].

In conclusion, there was no significant difference in recovery rate between the two methods and there was a positive correlation between the two methods. Hence, two techniques can be used to detect and quantify aflatoxin B1 in feed samples. However, there were variations among replicates in ELISA method which shows that this method is more applicable for screening purpose.

## Limitation

The only limitation of the research was shortage of project time to include field samples and other techniques for comparison.

## Data Availability

All collected data are presented in the manuscript in a summarized form.

## Abbreviations

AFB_1_: aflatoxin B_1_
ELISA: Enzyme-Linked Immunosorbent Assay
HPLC: high-performance liquid chromatography
RSD: relative standard deviation
SPE: solid phase extraction
USA: United States of America
